# Differences in Cholesterol Metabolism, Hepato-Intestinal Aging, and Hepatic Endocrine Milieu in Rats as Affected by the Sex and Age

**DOI:** 10.3390/ijms241612624

**Published:** 2023-08-10

**Authors:** Branka Šošić-Jurjević, Dieter Lütjohann, Svetlana Trifunović, Slađan Pavlović, Slavica Borković Mitić, Ljubiša Jovanović, Nataša Ristić, Ljiljana Marina, Vladimir Ajdžanović, Branko Filipović

**Affiliations:** 1 Department of Cytology, Institute for Biological Research “Siniša Stanković”—National Institute of Republic of Serbia, University of Belgrade, Bulevar Despota Stefana 142, 11060 Belgrade, Serbia; lanat@ibiss.bg.ac.rs (S.T.); negicn@ibiss.bg.ac.rs (N.R.); avlada@ibiss.bg.ac.rs (V.A.); brankof@ibiss.bg.ac.rs (B.F.); 2 Institute of Clinical Chemistry and Clinical Pharmacology, University Hospital Bonn, Venusberg-Campus 1, 53127 Bonn, Germany; dieter.luetjohann@ukbonn.de; 3 Department of Physiology, Institute for Biological Research “Siniša Stanković”—National Institute of Republic of Serbia, University of Belgrade, Bulevar Despota Stefana 142, 11060 Belgrade, Serbia; sladjan@ibiss.bg.ac.rs (S.P.); borkos@ibiss.bg.ac.rs (S.B.M.); 4 Department of Pathology and Medical Cytology, University Clinical Center of Serbia, Faculty of Medicine, University of Belgrade, Dr. Koste Todorovića 26, 11000 Belgrade, Serbia; jovanovicljubisa18@gmail.com; 5 National Centre for Infertility and Endocrinology of Gender, Clinic for Endocrinology, Diabetes and Metabolic Diseases, Faculty of Medicine, University of Belgrade, Koste Todorovića 6, 11000 Belgrade, Serbia; ljikka@yahoo.com

**Keywords:** sex differences, age, cholesterol metabolism, oxidative stress, liver, small intestine

## Abstract

Age and sex influence serum cholesterol levels, but the underlying mechanisms remain unclear. To investigate further, we measured cholesterol, precursors (surrogate synthesis markers), degradation products (oxysterols and bile acid precursors) in serum, the liver, jejunum, and ileum, as well as serum plant sterols (intestinal absorption markers) in male and female Wistar rats (4 and 24 months old). The analysis of histomorphometric and oxidative stress parameters (superoxide dismutase, catalase, glutathione-related enzyme activities, lipid peroxide, and protein carbonyl concentrations) in the liver and jejunum offered further insights into the age- and sex-related differences. The hepatic gene expression analysis included AR, ERα, and sex-specific growth hormone-regulated (Cyp2c11 and Cyp2c12) and thyroid-responsive (Dio1, Tbg, and Spot 14) genes by qPCR. We observed age-related changes in both sexes, with greater prominence in females. Aged females had significantly higher serum cholesterol (*p* < 0.05), jejunum cholesterol (*p* < 0.05), and serum plant sterols (*p* < 0.05). They exhibited poorer hepato-intestinal health compared with males, which was characterized by mild liver dysfunction (hydropic degeneration, increased serum ALT, *p* < 0.05, and decreased activity of some antioxidant defense enzymes, *p* < 0.05), mononuclear inflammation in the jejunal lamina propria, and age-related decreases in jejunal catalase and glutathione peroxidase activity (*p* < 0.05). Aged females showed increased levels of 27-hydroxycholesterol (*p* < 0.05) and upregulated ERα gene expression (*p* < 0.05) in the liver. Our study suggests that the more significant age-related increase in serum cholesterol in females is associated with poorer hepato-intestinal health and increased jejunal cholesterol absorption. The local increase in 27-hydroxycholesterol during aging might reduce the hepatoprotective effects of endogenous estrogen in the female liver.

## 1. Introduction

Homeostasis of cholesterol is achieved by tight regulation of its uptake, synthesis, and degradation to bile acids before excretion via bile. Although all mammalian cells can synthesize cholesterol to a small extent, the liver is the main site of its endogenous synthesis and degradation [1]. In addition, the small intestine contributes greatly to the regulation of dietary exogenous and endogenous biliary cholesterol levels and is the second largest site of endogenous cholesterol synthesis after the liver [2].

Obesity, poor diet, and associated metabolic disorders are the most important independent risk factors for the development of atherosclerotic cardiovascular and liver disease, but age and sex also contribute significantly [3,4]. In men, atherosclerotic cardiovascular disease (ASCVD) typically manifests 10 to 15 years earlier than in women, and traditional risk factors appear to contribute to ASCVD in a sex-specific manner [5]. Moreover, both preclinical and clinical studies indicate sex differences in plaque morphology [5,6]. Prevention strategies place great importance on the lowering of circulating cholesterol. In blood, free and esterified cholesterol is mainly transported in pro-atherogenic, apolipoprotein B (apoB)-containing non-high-density lipoproteins (non-HDLs), low-density lipoproteins (LDLs), and further triglyceride-rich lipoproteins such as very low-density lipoproteins (VLDLs), intermediate-density lipoproteins (IDLs), chylomicrons, and chylomicron remnants [7,8]. A growing number of reports in the literature suggest that non-HDL-C is a better lipoprotein marker for any atherosclerotic disease than LDL-C or ApoB in humans [9,10], Although differences between men and women in plasma lipid profiles, hepatic lipid metabolism, and response to pharmaceutical approaches to lower cholesterol have been described [11], the molecular mechanisms underlying these sex differences are still unclear.

The liver is a sexually dimorphic organ, which exhibits differential effects of sex hormones on metabolic pathways, gene transcription, and susceptibility to liver disease [12]. The effects of sex steroids on the regulation of gene expression in the liver are largely mediated by pituitary GH [13]. Chronic liver disease is more common and severe in males of reproductive age, whereas postmenopausal women are more likely to develop nonalcoholic steatohepatitis and advanced fibrosis, indicating the protective role of estrogen in the liver [14]. Oxidative stress, a common feature of chronic liver disease, is generally higher in males [15] but may increase with age in females [16]. In addition, oxidative stress plays an important role in the early phase of intestinal damage [17]. Epidemiological studies suggest that women predominate in functional gastrointestinal disorders [18]. Moreover, epidemiological studies suggest women’s predominance in functional gastrointestinal disorders such as pain/discomfort symptoms, irritable bowel syndrome, and constipation, indicating the effect of sex hormones on their pathogenesis [18,19,20]. However, in the context of human hepato-intestinal disorders, it is difficult to distinguish the effects of biological sex from the impact of sex-specific socioeconomic and cultural differences.

Rodent models of aging exhibit sex differences in many organs and physiological functions, as well as sex differences in aging and age-related diseases [21]. Compared to humans, rodents have similar cholesterol metabolic pathways; however, they exhibit higher synthesis and degradation rates, along with faster clearance. Additionally, they primarily transport their serum cholesterol in HDL particles [22]. Furthermore, aging females in middle age (12–16 months) do not experience a dramatic drop in estrogen similar to the human menopause but face a progressive and gradual age-related decline in their hormonal and reproductive potential [23].

Considering all of the aforementioned data, the objective of this study was to investigate the sex differences in specific pathways of cholesterol metabolism, specifically examining serum, the liver, and small intestine, and assessing how these pathways are influenced by advanced age in rats on a normal diet. Furthermore, we aimed to explore potential associations between changes in cholesterol metabolism, hepato-intestinal health, and variations in the hepatic endocrine environment. Finally, we aimed to gain insights into potential sex-specific mechanisms that could impact overall metabolic health in aging individuals. These findings may contribute to a deeper understanding and the development of personalized, sex-specific prevention and treatment strategies.

## 2. Results

### 2.1. Concentration of Total Cholesterol in Serum, the Liver, Jejunum, and Ileum

**Figure 1:**
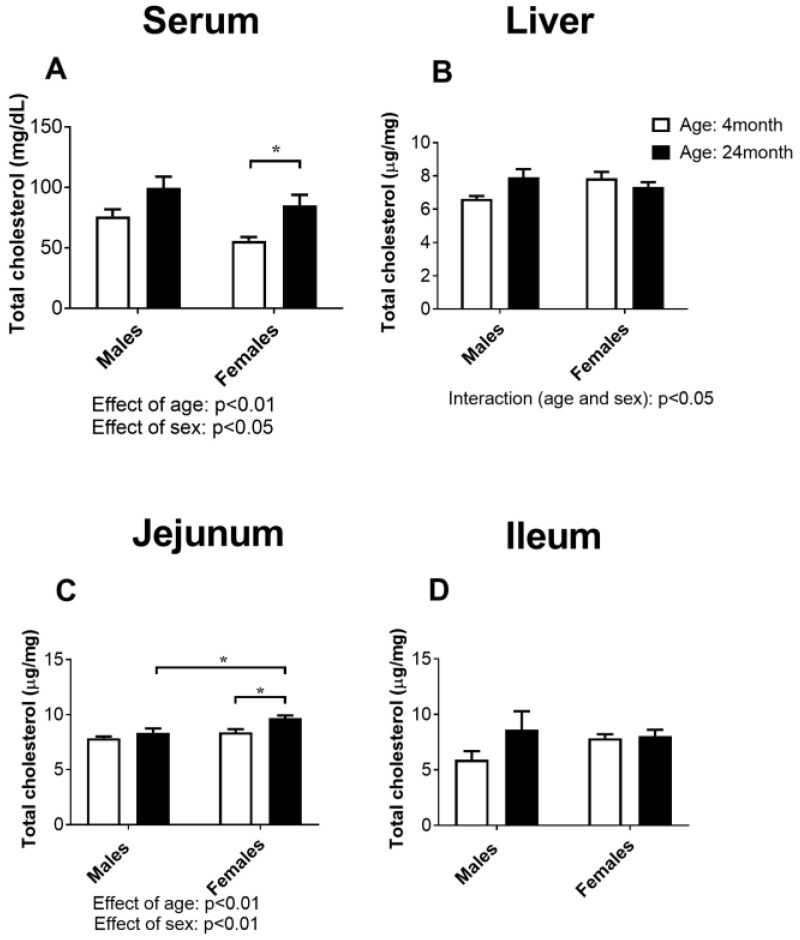
Concentration of total cholesterol in the serum (**A**), liver (**B**), jejunum (**C**), and ileum (**D**) of young adult (4-month-old) and old-aged (24-month-old) male and female rats (*n* = 6/group). The data are presented as the mean ± SEM, *n* = 6/group, two-way ANOVA followed by Tukey’s post hoc test, * *p* < 0.01.

The results of the total cholesterol in the serum, liver, jejunum, and ileum of the rats are shown in Figure 1. The value of total cholesterol in serum was increased in old-aged rats of both sexes (effect of age: *p* < 0.01, two-way ANOVA), while females had lower total cholesterol compared to males of the same age (effect of sex: *p* < 0.05, two-way ANOVA). Post-hoc comparison confirmed that the concentration of total cholesterol in serum increased (*p* < 0.05, Tukey test) only in old-aged females (Figure 1A).

Liver total cholesterol was not significantly different between the experimental groups irrespective of age or sex (Figure 1B), but the effect of both age and sex was slightly different (effect of interaction: *p* < 0.05).

Total cholesterol in the jejunum was increased (effect of age: *p* < 0.01, two-way ANOVA), but only in females (effect of sex: *p* < 0.01, two-way ANOVA; Figure 1C), which was confirmed by the post hoc analysis (*p* < 0.05, Tukey’s test) (Figure 1C).

The statistical analysis did not confirm any significant difference between the experimental groups in the concentration of total cholesterol in the ileum (Figure 1D).

### 2.2. Concentration of HDL-Cholesterol and Non-HDL-Cholesterol in Serum

**Figure 2:**
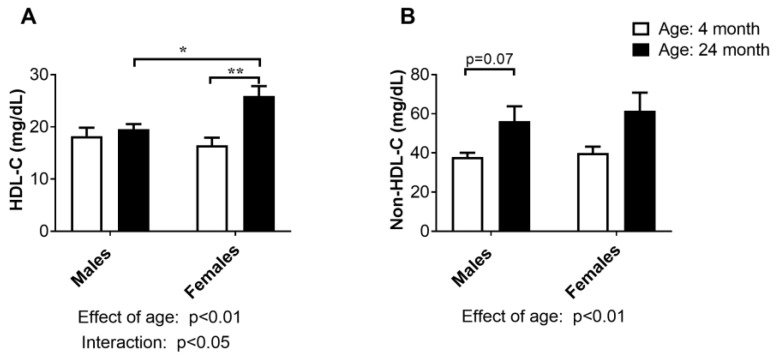
Concentration of cholesterol content in high-density lipoproteins (HDL-C; (**A**)) and non-high-density (non-HDL-C; (**B**)) lipoprotein cholesterols in the serum of young adult (4-month-old) and old-aged (24-month-old) male and female rats. The data are presented as the mean ± SEM, *n* = 6/group; * *p* < 0.05 and ** *p* < 0.01, two-way ANOVA followed by Tukey’s post hoc test.

HDL-C concentration in serum increased with advanced age (effect of age: *p* < 0.01, two-way ANOVA), as confirmed by the post hoc analysis for the females (*p* < 0.01, Tukey’s test, Figure 2A). The interaction between age and sex in HDL concentration in serum (*p* < 0.05, two-way ANOVA) suggests that the effect of age on HDL levels differs between males and females, and old-aged females had higher values of this parameter than males of the same age (*p* < 0.05; Tukey’s test; Figure 2A). Non-HDL-C encompasses all atherogenic cholesterols. The non-HDL concentration in the serum increased in both sexes of advanced age (effect of age: *p* < 0.01, two-way ANOVA, Figure 2B), and this change seemed to be more prominent in old-aged males (*p* = 0.07, Tukey’s test, Figure 2B).

### 2.3. Concentration of Plant Sterols in Serum

Plant sterols are compounds with a similar chemical structure and biological functions as cholesterol. In the small intestine, they are transported to the lumen, but the rate of absorption is much lower compared with cholesterol; therefore, cholesterol absorption can be assessed by measuring non-cholesterol sterols in serum.

**Figure 3:**
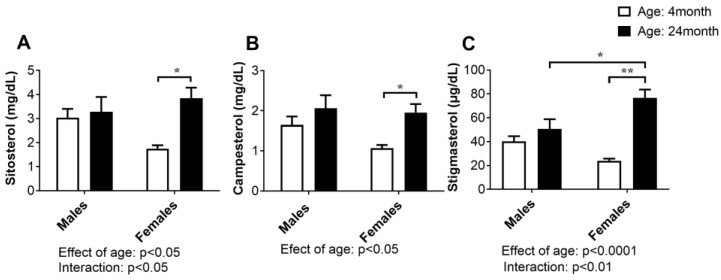
Concentration of plant sterols: sitosterol (**A**), campesterol (**B**), and stigmasterol (**C**) in the serum of young adult (4-month-old) and old-aged (24-month-old) male and female rats. The data are presented as the mean ± SEM, *n* = 6/group; * *p* < 0.05, ** *p* < 0.01, two-way ANOVA followed by Tukey’s post hoc test.

Age- and sex-specific differences in the concentration of the analyzed plant sterols, sitosterol, campesterol, and stigmasterol, are summarized in Figure 3. Two-way ANOVA showed a significant effect of age for three studied parameters: sitosterol (*p* < 0.05), campesterol (*p* < 0.05), and stigmasterol (*p* < 0.0001), and the effect of age and sex (interaction) in the case of sitosterol (*p* < 0.05) and stigmasterol (*p* < 0.01). Post hoc comparisons confirmed that the age-related increase in all the above parameters was significant only in the females (*p* < 0.05, Tukey’s test).

### 2.4. Concentration of Cholesterol Precursors in Serum, Liver, Jejunum and Ileum

**Figure 4:**
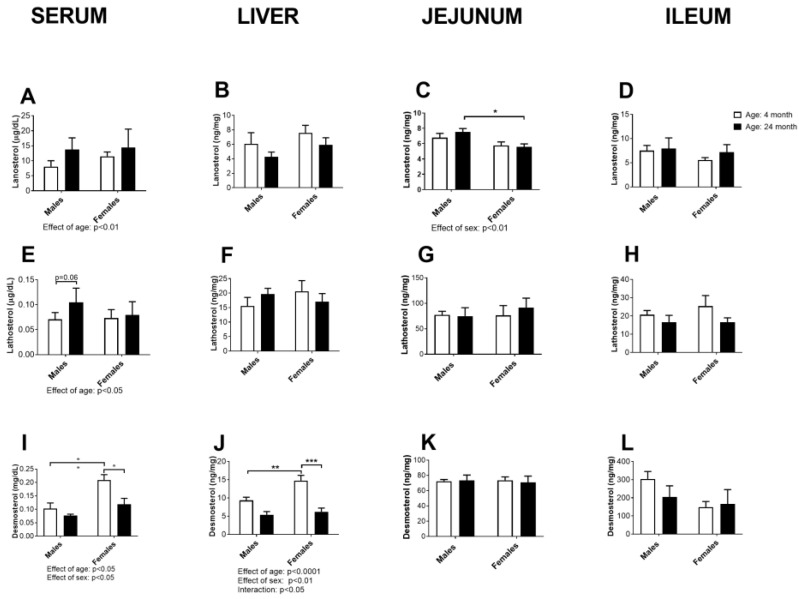
Concentration of cholesterol biosynthetic precursors: lanosterol (**A**–**D**), lathosterol (**E**–**H**) and desmosterol (**I**–**L**) in the serum, liver, jejunum, and ileum of young adult (4 months old) and old-aged (24-month-old) male and female rats (*n* = 6/group). The data are presented as the mean ± SEM, two-way ANOVA followed by Tukey’s post hoc test, * *p* < 0.05, ** *p* < 0.01, and *** *p* < 0.001.

The results of the concentration of the biosynthetic precursors of cholesterol, lanosterol, lathosterol, and desmosterol in serum and in the organs most important for cholesterol biosynthesis are summarized in Figure 4.

The serum concentrations of lanosterol and lathosterol were slightly increased in the age groups of both sexes (effect of age: *p* < 0.01 and *p* < 0.05, respectively, two-way ANOVA), without being accompanied by a significant increase in these parameters in the liver or small intestine (Figure 4A–H). Males had higher concentrations of lanosterol in the jejunum in comparison to females (effect of sex: *p* < 0.01, two-way ANOVA), which was also confirmed for older groups by post hoc comparisons (*p* < 0.05, Tukey’s test, Figure 4C).

However, the serum desmosterol concentration decreased in an age- and sex-dependent manner (effect of age: *p* < 0.01 and effect of sex: *p* < 0.01, two-way ANOVA: Figure 4I): young adult females had higher (*p* < 0.05, Tukey’s test) serum desmosterol concentrations than the males (Figure 4I). The obtained age and sex differences were also evident in the liver (effect of age: *p* < 0.0001 and effect of sex: *p* < 0.01, interaction: *p* < 0.05, two-way ANOVA); the young adult females had higher desmosterol concentrations than the males (*p* < 0.01, Tukey’s test), and age-related decline of hepatic desmosterol was significant only in the female rats (*p* < 0.001, Tukey test). We observed a sex-specific decrease in serum lanosterol levels in the jejunum (*p* < 0.05, Tukey’s test). However, no significant changes with respect to age were observed for the examined precursors in the jejunum.

### 2.5. Liver Weight, Histology, and Concentration of Aminotransferases in Serum

**Figure 5:**
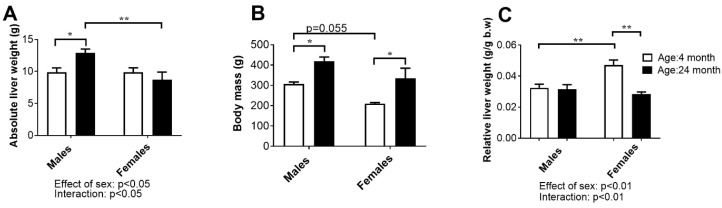
The absolute liver weight (**A**), body mass (**B**), and relative (**C**) liver weight of young adult (4-month-old) and old-aged (24-month-old) adult male and female rats. The data are presented as the mean ± SEM, *n* = 6/group; * *p* < 0.05 and ** *p* < 0.01, two-way ANOVA followed by Tukey’s post hoc test.

The results of mean absolute liver weight, body mass, and relative liver weight are shown in Figure 5.

Two-way ANOVA showed a significant sex difference (*p* < 0.05) in absolute liver weights: males had higher values for these parameters than females, and the difference was more significant between the age groups (*p* < 0.01, Tukey’s test). Moreover, the age-related increase in liver weight was found only in males at older ages (*p* < 0.05, Tukey’s test) (Figure 5A).

The average body mass of the young adult male rats was slightly higher (*p* = 0.055, two-way ANOVA) compared to the female rats of the same age. In both sexes, the effect of age was statistically significant (*p* < 0.05): the body mass of the old males was higher than that of the young adult males and females compared with the corresponding groups of young adults (Figure 5B).

The relative liver weight (liver-to-body-weight ratio) was lower in young adult females than in males (*p* < 0.01, two-way ANOVA and Tukey’s test), consistent with the difference in body mass. However, the effect of age was different between the sexes (interaction: *p* < 0.01, two-way ANOVA); this parameter did not change significantly with age in males, while in females it decreased (*p* < 0.01, Tukey’s test) (Figure 5C).

**Figure 6:**
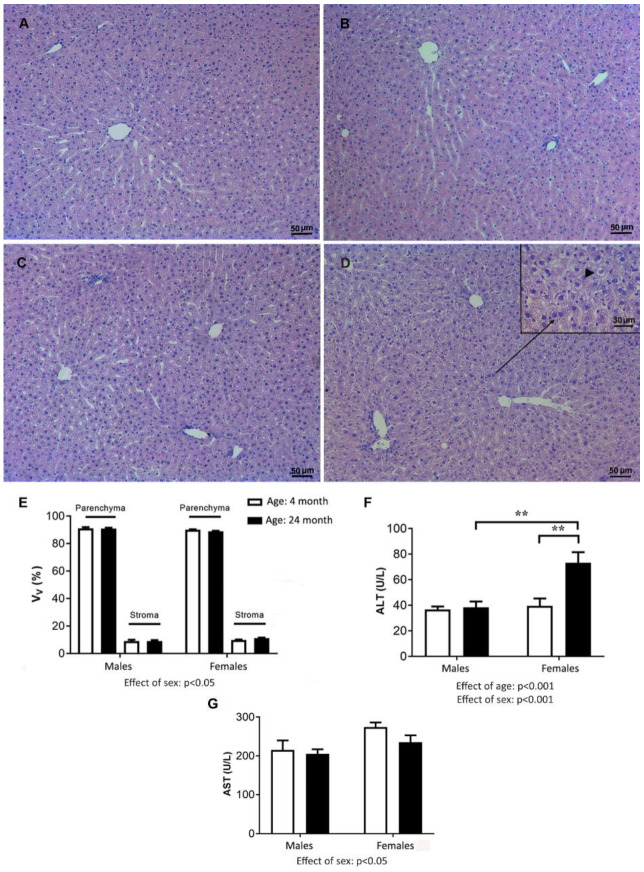
Representative micrographs of hematoxylin-and-eosin-stained liver sections from young (4-month old) and old-aged (24-month-old) male (**A**,**B**) and female rats ((**C**,**D**); inset display area with marked hydropic degeneration of hepatocytes at higher magnification, black arrow point on hepatocyte swelling), respectively; the relative volume density of liver parenchyma (hepatocytes) and stroma (including sinusoids, portal tracts, bile ducts, central veins, and others) (**E**); serum concentrations of alanine (ALT; (**F**)) and aspartat (AST; (**G**)) aminotransferase in the serum of the experimental groups; the data are presented as the mean ± SEM, *n* = 6/group, two-way ANOVA followed by Tukey’s post hoc test, ** *p* < 0.01.

The liver histology of the young males was characterized by normal lobule morphology and the usual arrangement of hepatocytes around the central vein (Figure 6A,B). The livers of the old animals showed normal architecture but also some degree of hydropic degeneration, manifested as hepatocyte hypertrophy and ballooning, which was more pronounced in old-aged females than in males (Figure 6C,D). The morphometric analysis confirmed that the stromal–parenchymal indices of the liver did not change significantly with aging (Figure 6E). Histological assessment of the liver sections using Sirius Red showed no abnormalities in tissue structure in either the young or aged animals of both sexes (Supplementary Figure S1). Statistical analysis of the difference in the mean concentration of ALT in serum between the experimental groups using two-way ANOVA showed a significant effect of sex and age (*p* < 0.001) as well as the interaction between these two factors (*p* < 0.001). Post hoc analysis confirmed that ALT was higher (*p* < 0.01) in the aged female group than in all other experimental groups (Figure 6F). In contrast, analysis of differences in AST using a two-way ANOVA revealed an influence of sex (*p* < 0.05): females had a higher AST compared to males regardless of age (Figure 6G).

### 2.6. Oxidative Stress Parameters in the Liver

The results of the studied parameters of oxidative stress in the liver of young adult and old male and female rats are shown in Table 1.

Our results show a significant influence of age (*p* < 0.05, two-way ANOVA) on the parameters of oxidative defense and oxidative damage in both sexes. Specifically, in the liver of the male rats, the activity of SOD and the concentrations of GSH and PCO were significantly lower in the 4-month-old rats compared with the 24-month-old rats (*p* < 0.05, Tukey’s test), while the activities of CAT and GR were higher (*p* < 0.05, Tukey’s test). In females, the activities of SOD and CAT were lower (*p* < 0.05, Tukey’s test), while the concentration in the SH group was higher (*p* < 0.05, Tukey’s test) (Table 1).

Regarding the effect of sex (*p* < 0.05, two-way ANOVA), we obtained significantly higher activities of SOD, CAT, GSH-Px, and GR and concentrations of LPO (*p* < 0.05, Tukey’s test) and decreased activity of GST and concentrations of GSH and PCO (*p* < 0.05) in the 4-month-old females compared with the 4-month-old males. In the 24-month-old animals, the activity of CAT and the concentration of GSH were significantly decreased, while the activity of GSH-Px and the concentration of LPO were increased in the livers of the female rats compared to the male rats (*p* < 0.05, Tukey’s test) (Table 1).

### 2.7. Hormone-Responsive Gene Expressions in the Liver

**Figure 7:**
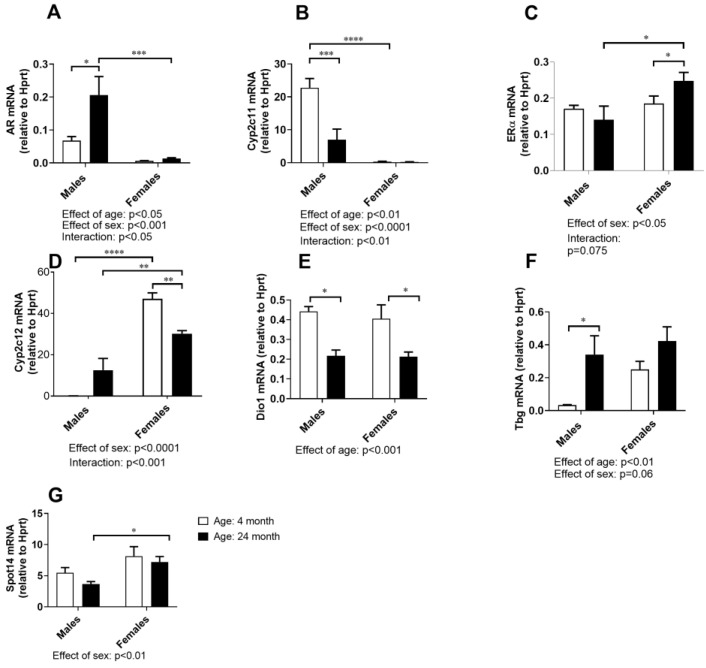
mRNA expression of AR (**A**), Cyp2c11 (**B**), ERα (**C**), Cyp2c12 (**D**), Dio1 (**E**), Tbg (**F**), and Spot14 (**G**) in the liver of young adult (4-month-old) and old-aged (24-month-old) male and female rats; the data are presented as the mean ± SEM, *n* = 6/group; two-way ANOVA followed by Tukey’s post hoc test, * *p* < 0.05, ** *p* < 0.01, *** *p* < 0.001, and **** *p* < 0.0001.

The mRNA expression analysis of hormone-dependent genes in the liver included (Figure 7): the sex steroid receptors most abundant in the liver (AR and Erα), the typical male- and female-specific GH-regulated genes (Cyp2c11 and Cyp2c12, respectively), and thyroid-responsive genes (Dio 1, Tbg, and Spot 14). The gene expressions of AR and Cyp2c11 were highly male specific (effect of sex: *p* < 0.01 and *p* < 0.0001, respectively, two-way ANOVA) and age dependent (age effect: *p* < 0.05, and *p* < 0.01, respectively; interaction: *p* < 0.01, and *p* < 0.05, two-way ANOVA); AR gene was upregulated (*p* < 0.05, Tukey’s test), whereas Cyp2c11 was downregulated in the liver of old-aged males (*p* < 0.01, Tukey’s test) (Figure 7A,B). The ERα and Cyp2c12 gene expressions were higher in the female livers than in the male livers (effect of sex: *p* < 0.01, and *p* < 0.0001, respectively, two-way ANOVA), and the effect of sex was age-dependent (interaction: *p* = 0.075 and *p* < 0.01, respectively, two-way ANOVA); ERα mRNA levels were higher in older females than males of the same age (*p* < 0.05, Tukey’s test), while age-related downregulation of Cyp2c12 was more prominent (*p* < 0.05, Tukey’s test) than the increase in ERα mRNA levels (Figure 7C,D).

Possible sex- and age-related differences in the gene expression of well-known thyroid hormone (TH)-responsive genes in the liver were also investigated, considering the role of TH in cholesterol homeostasis and the possible sex dimorphism of TH-dependent gene expressions. Age-dependent downregulation of the positively regulated TH-responsive gene Dio 1 (*p* < 0.05, Figure 7E) and increased expression of the negatively-regulated gene Tbg (*p* < 0.01, Figure 7F) were found in the livers of old vs. young adult rats of both sexes, with the latter being more pronounced in males than in females (effect of sex: *p* = 0.06, two-way ANOVA; *p* < 0.05 old vs. young males, Tukey’s test, Figure 7F), indicating TH deficiency in the livers of both sexes. For the gene expression of the Spot 14 gene, the effect of sex was most evident: females had higher expression than the males (effect of sex: *p* < 0.01, two-way ANOVA, *p* < 0.05, Tukey’s test, Figure 7G), while the age-related decline in the expression of this gene was not as evident as for the other examined TH-responsive genes.

### 2.8. Histology of the Jejunum

**Figure 8:**
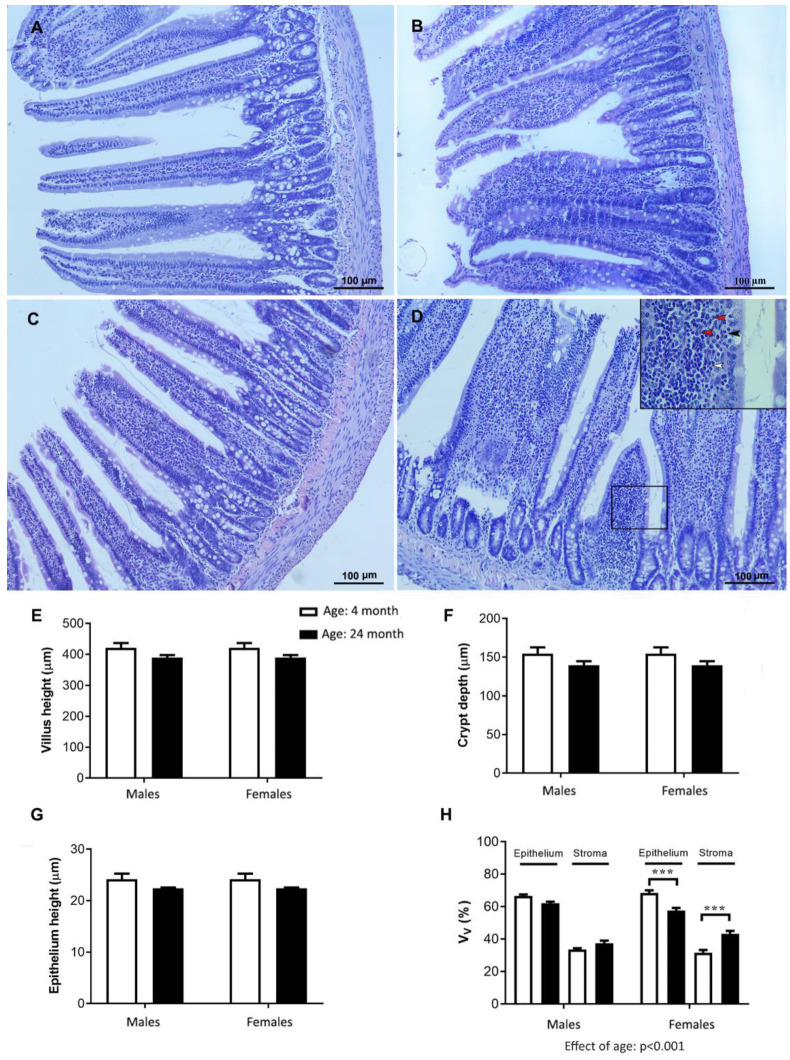
Representative micrographs of hematoxylin-and-eosin-stained jejunum sections from young adult (4-month-old) and old-aged (24-month-old) male (**A**,**B**) and female ((**C**,**D**); the inset displays stromal and intraepithelial inflammation; the black arrowheads point to intraepithelial lymphocytes, the white arrowheads point to lymphocytes, the while red arrowheads point to plasma cells) rats, respectively; morphometric analysis of jejunal mucosa: villus height (**E**), crypt depth (**F**), epithelium height (**G**), and the relative volume density (V_V_; (**H**)) of the epithelium and stroma in the villi of the experimental groups; the data are presented as the mean ± SEM, *n* = 6/group, two-way ANOVA followed by Tukey’s post hoc test, *** *p* < 0.001.

The results obtained for cholesterol metabolism prompted us to examine the jejunal sections for possible age- and sex-related histopathological and morphometric changes (Figure 8). The jejunal villi had mainly a finger-like morphology in the young adult rats in both sexes and in the old-aged males (Figure 8A–C). The average V/C ratio was 3:1. In the old-aged females, the villi appeared slightly shorter and blunter, with the constriction of the epithelium over the stroma. In addition, it is characterized by a stromal and intraepithelial inflammatory infiltrate (mononuclear inflammation consisting of lymphocytes and plasma cells) (Figure 8D).

The morphometric analysis showed that the average height of the jejunal villi, the height of the epithelium, and the depth of the crypt did not significantly decrease with age, either in the males or females (Figure 8E–G). The average V/C ratio remained unaltered. Within the villi, the relative volume density of the epithelium decreased, while the relative volume density of the stroma increased with age (effect of age, *p* < 0.001, two-way ANOVA; Figure 8H). The observed changes were more pronounced in the female animals. The relative volume density of the epithelium decreased (*p* < 0.001; Tukey’s post hoc test) only in the old-aged females, whereas the relative volume density of the stroma increased (*p* < 0.001; Tukey’s post hoc test) compared with the values obtained for the young adult females (Figure 8H).

### 2.9. Oxidative Stress Parameters in the Jejunum

The results for the oxidative stress parameters in the jejunum of the studied animals are summarized in Table 2.

The two-way ANOVA analysis showed an age-related decrease (*p* < 0.05) in the activities of glutathione-related enzymes (GSH-Px, GR, and GST) in the jejunum of the 24-month-old rats compared to those of the 4-month-old rats (post hoc Tukey’s test, *p* < 0.05) regardless of sex. Only the 24-month-old female rats had significantly reduced activity of CAT, which was compared to the 4-month-old female rats (post hoc Tukey’s test, *p* < 0.05) (Table 2).

The effect of sex was significant (two-way ANOVA; *p* < 0.05), showing that females had significantly higher activity of CAT compared to with the males compared to age-matched groups (post-hoc Tukey’s test, *p* < 0.05) and higher concentrations of GSH (post-hoc Tuckey’s test, *p* < 0.05), but lower activity of GSH-Px and GST and lower concentrations of SH groups and LPO (post-hoc Tukey, *p* < 0.05) in the jejunum (Table 2).

### 2.10. Concentration of Cholesterol Degradation Intermediates in Serum, the Liver, Jejunum, and Ileum

**Figure 9:**
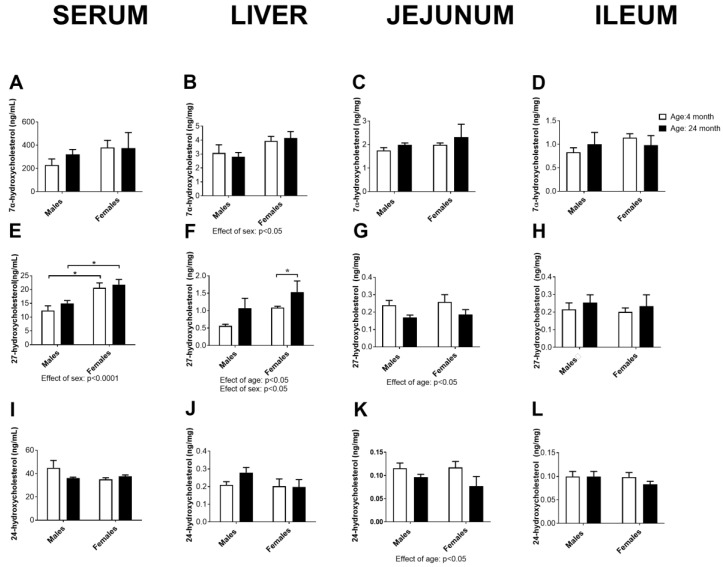
Concentration of 7α-hydroxycholesterol (**A**–**D**), 27-hydroxycholesterol (**E**–**H**), and 24-hydroxycholesterol (**I**–**L**) in the serum, liver, jejunum, and ileum of young (4 months old) and old (24 months old) adult male and female rats (*n* = 6/group). The data are presented as te mean ± SEM, two-way ANOVA followed by Tukey’s post hoc test, * *p* < 0.05.

The concentration of the main cholesterol degradation intermediates leading to the formation of bile acid, 7α-hydroxycholesterol, 27-hydroxycholesterol, and 24-hydroxycholesterol, are summarized in Figure 9. The results obtained indicate a sex difference in the hepatic concentrations of 7α- and 27-hydroxycholesterol (*p* < 0.05, two-way ANOVA): females had higher hepatic concentrations of these parameters than males of the same age, which was associated with the same effect in serum in the case of 27-hydroxycholesterol (effect of sex: *p* < 0.0001, two-way ANOVA; *p* < 0.05, Tukey’s test). For hepatic 27-hydroxycholesterol, the effect of age was also significant (*p* < 0.05, two-way ANOVA), which was confirmed by post hoc comparisons only in females (*p* < 0.05, Tukey’s test).

Age-related decreases in 27- and 24-hydroxycholesterol concentrations in the jejunum were also evident (*p* < 0.05, two-way ANOVA), indicating somewhat decreased degradation of cholesterol in this organ with age.

### 2.11. Gene and Immunohistochemical Analysis of CYP27A1 in the Liver

**Figure 10:**
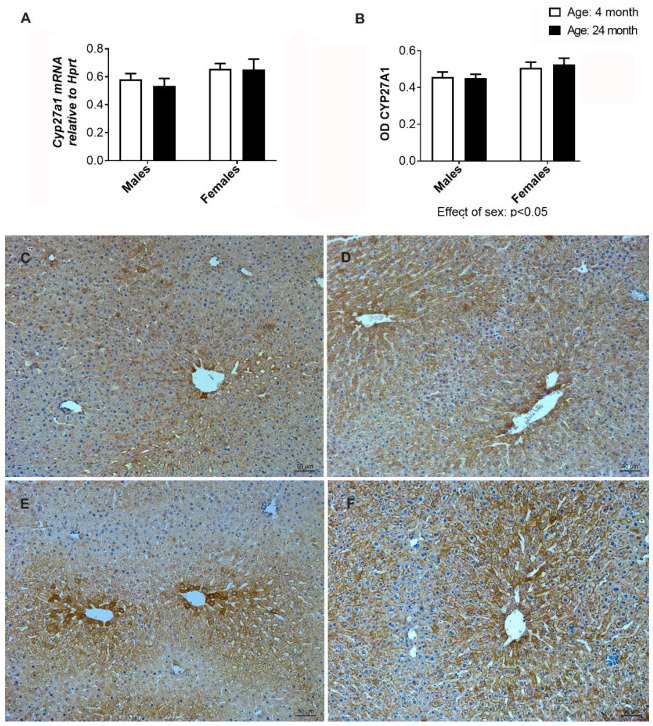
mRNA expression of Cyp27a1 (**A**) and optical density (OD) of CYP27A1 (**B**) in the liver, and the representative micrographs of immunohistochemical staining showing localization of CYP27A1 in the livers of the young (4-month-old) and old-aged (24-month-old) male (**C**,**D**) and female (**E**,**F**) rats, respectively.

Having in mind the elevated levels of 27-hydroxycholesterol in the liver of aged females, we aimed to further investigate the gene expression and immunohistochemical analysis of CYP27A1. The mRNA expression analysis of Cyp27a1 showed no significant difference (Figure 10A), whereas immunostaining analysis of CYP27A1 proteins showed differences between the experimental groups (Figure 10B–F). In females, the intensity of CYP27A1 immunostaining was higher than in males, regardless of age, with statistical significance (*p* < 0.05). However, when young adult females were compared with old-aged females, a difference in the distribution pattern of CYP27A1 was noted. In young adults, the most intense immunostaining was visible in the centrilobular hepatocytes (Figure 10E). In the old-aged females, immunopositivity was more evenly distributed across hepatocytes ranging from the centrilobular to the mid-lobular regions (Figure 10F).

**Figure 11:**
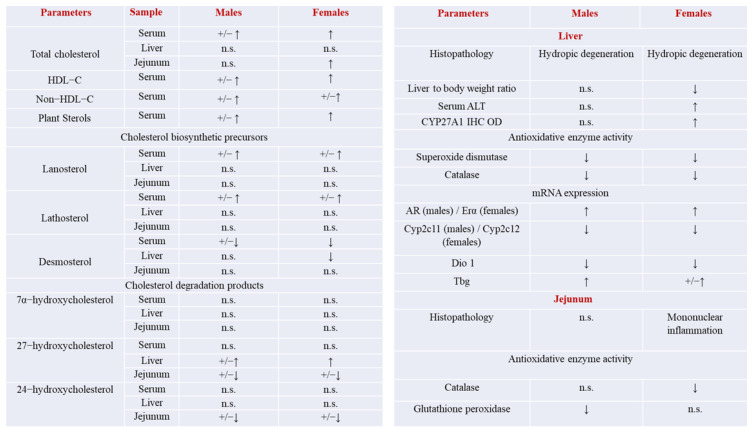
Summary of findings obtained from the comparison of the young (4-month-old) and old-aged (24-month-old) adult male or female rats (*n* = 6/group). +/−↑ and +/−↓ represent the significance evaluated by two-way ANOVA analysis (effect of age: *p* < 0.05 and less), and ↑ and ↓ represent the results of the post hoc Tukey’s assay, while n.s. indicates non-significant changes.

The findings obtained for the effects of age on the examined parameters of cholesterol metabolism (left panel) in serum, the liver, and jejunum, as well as the most important histopathological and antioxidative enzyme activity changes in the liver and jejunum (right panel) of the male and female rats, are summarized in Figure 11.

## 3. Discussion

The aim of this study was to investigate sex differences in cholesterol metabolism and hepato-intestinal aging in rats fed a normal diet to mitigate the impact of an unhealthy high-fat diet.

Initially, we demonstrated an age-related increase in serum total cholesterol levels in both sexes, but a post hoc comparison confirmed that the serum total cholesterol concentrations increased only in the old-aged females. Elevated cholesterol levels and an unfavorable lipid profile are known modifiable risk factors for cardiometabolic disease. In humans, an age-related increase in serum cholesterol levels is observed, and the association between age and serum cholesterol levels may vary between males and females [24]. In general, women’s cholesterol levels often increase after the menopause and may approach or even exceed those of men [25]. Importantly, our results showed that elevated serum cholesterol levels were associated with an increase in total cholesterol in the jejunum in both males and females. However, this change proved to be statistically significant only in the females. In addition, the serum levels of phytosterols were found to increase with age in both sexes, although statistical significance was again found only in female rats. The absorption efficiency of plant sterols in the small intestine is significantly lower than that of cholesterol; therefore, their concentrations in the blood can serve as reliable indicators of cholesterol absorption in the intestine [26]. The transport of cholesterol and plant sterols by enterocytes is an active and highly selective process, mediated mainly by the protein Niemann-Pick C1-like 1 (NPC1L1). Conversely, the proteins adenosine triphosphate (ATP)-binding cassette (ABC) transporters Abcg5 and Abcg8 play critical roles as important efflux transporters for cholesterol and plant sterols in the intestine [27]. Gene and protein expression of the sterol transporter NPC1L1 is higher in the jejunum than in the ileum in both rats and mice [28]. Ultimately, no significant changes in markers of cholesterol synthesis in the jejunum suggest that changes in cholesterol transport are the major underlying mechanism for the increase in serum cholesterol in older female animals. This assumption is supported by the results of Duan et al. [29], who showed that aging in mice suppresses the expression levels of jejunal and ileal Abcg5 and Abcg8 and upregulates the expression levels of jejunal Npc1l1.

Next, we analyzed age- and sex-related differences in HDL-C and non-HDL-C concentrations in serum. Wild-type rats carry most of their cholesterol in HDL and are therefore not prone to atherosclerosis development [28]. Our results showed that HDL-C increased with age in both sexes, but a post hoc comparison confirmed an increase only in the old-aged females. On the other hand, atherogenic non-LDL-C was more prominently increased with advanced age in males, which generally aligns with findings in rodents and humans [30,31,32,33,34].

Analysis of liver cholesterol precursors revealed age-related increases in lanosterol and lathosterol, whereas hepatic desmosterol showed a significant decrease according to the post hoc analysis. It is important to note that liver total cholesterol remained unchanged. These results and the fact that the total hepatic cholesterol remained unchanged strongly suggest that the age-related increase in serum cholesterol levels in rats is not primarily due to changes in hepatic cholesterol biosynthesis or a decrease in hepatic catabolism. The age-related decrease in desmosterol in female animals could be a consequence of specific changes in the post-lanosterol Bloch pathway. However, it is also possible that desmosterol, as an immediate precursor of cholesterol, is also a substrate for CYP27A1, which has been demonstrated in vitro [35].

To evaluate age-related changes in the liver, we used physiological, histopathological, and oxidative stress analyses. We demonstrated an age-related decrease in liver-to-body-weight ratio and increased serum ALT levels in older females. However, the microscopic changes were subtle and manifested primarily as hydropic degeneration, which was seen in both sexes, although it was slightly more common in older females. Wild-type Wistar rats are not naturally prone to developing fatty liver, and steatosis is usually induced by providing a special fatty diet containing cholesterol [36]. While our H&E and Sirius Red analysis did not confirm the presence of steatosis and fibrosis in the livers of the aged animals, further exploration through the application of the Oil red—O staining technique is required to gain deeper insights into the lipid storage changes in the aged liver. The data obtained suggest that liver function is impaired with age in females, though the histological and functional changes do not correlate directly, which is consistent with the findings of previous studies [37]. Further histochemical analysis of beta-galactosidase could provide insights into cellular senescence in the liver, which may lead to impaired liver function. Cao et al. [38] showed that aging in mice is accompanied by changes in the expression of genes in the liver that are involved in age-related liver pathologies.

When considering oxidative stress, we cannot just look at a single parameter but must look at the system as a whole [15]. The collected data on oxidative stress in the liver, especially the lower activity of SOD, catalase, and most glutathione-related enzymes, together with higher PCO levels, suggest that the livers of young adult males are exposed to greater oxidative stress compared to females. These results are consistent with previous reports by Chen et al. [39]. As expected, the livers of the aged rats, regardless of sex, generally exhibited increased levels of oxidative stress and a general decrease in the activity of the enzymes studied. However, the aged female livers showed, a marked decrease in catalase activity and an increase in hepatic oxidative damage (LPO) compared with the aged male livers. Malondialdehyde (MDA) is a well-characterized product of lipid peroxidation [17]. Overall, our results strongly suggest that the aged female livers are in poorer health than those of healthy aged males.

We next examined the sex difference in age-related changes in the endocrine environment of the liver. This was conducted indirectly by analyzing the hormone-responsive gene expressions. We assessed one of the most abundant sex steroid receptors in the liver (AR and ERα), sex-specific GH-regulated genes (Cyp2c11 for males and Cyp2c12 for females) [13], and thyroid hormone-responsive genes (Dio 1, Tbg, and Spot 14). Thyroid disease is commonly associated with dyslipidemia and liver pathology, and it appears that thyroid hormones exhibit sex-specific regulation of several target genes in the liver [40]. The increasing expression of AR in the liver of males and the ERα in the liver of females with age may suggest that the liver undergoes adaptive changes to maintain tissue sensitivity when hormone concentrations become scarce. ERα is the most abundantly expressed form of ER in the liver, and impaired ERα function is associated with obesity and metabolic dysfunction in humans and rodents [41]. ERα was expressed in the liver of males, consistent with previous findings [42]. The male and female GH-specific genes Cyp2c11 and Cyp2c12 [13] were downregulated in aged animals, suggesting lower GH availability. In addition, the presence of the expression of female-specific GH-regulated Cyp2c12 in aged males indicates a more “female-like“ GH secretion profile in aged males [43]. Dio1 gene expression was downregulated with advanced age in both sexes [40], while Tbg and Spot 14 gene expressions were also sex dependent as well, in line with previous reports [44,45]. Overall, the gene expression profiles of all genes studied indicate decreased hormone availability in the aged liver of both sexes.

Histomorphometric analysis of the jejunum revealed minor age-related changes in intestinal morphology, consistent with the results of other authors [46]. More importantly, we observed signs of chronic mononuclear inflammatory processes in the jejunum mucosa of the older females. Research linking the immune system, systemic chronic inflammation, and the gut microbiome to endocrine functions is still in its infancy, whereby bacteria can respond to host hormones (estrogens) and vice versa [47]. Recently, an immune-mediated pathway linking the gut-microbiota-derived metabolite propionate to intestinal NPC1L1 expression and activity has been described [48]. Several studies have confirmed the link between bile acids, sex hormones, and gut microbiota composition [49]. Given the observed increase in liver 27-hydroxycholesterol, it is reasonable to speculate that the bile acid composition may be altered in older females. Such changes in bile acid composition could possibly contribute to the inflammatory process in the jejunum.

We also examined oxidative stress in the jejunum. Interestingly, the data obtained, indicating higher catalase and most glutathione-related enzymes, together with higher concentrations of SH and LPO, show that males have lower antioxidant defenses and higher oxidative-stress-induced damage in the jejunum compared to females, regardless of age. When we focused on age-related changes specifically in females, we observed a decrease in CAT activity when we compared young adult females with older females. In addition, there was a decrease in GSH-Px activity when comparing aged males and females, which is consistent with previous findings [50]. It is important to note that CAT and GSH-Px play crucial roles in scavenging hydrogen peroxide [51], which has been implicated in mechanisms related to the intestinal transport dysfunction of ions and inflammation onset in rodent colitis models [52]. All factors responsible for oxidative stress are directly or indirectly involved in the immune system’s defense mechanism [53]. Further studies are needed to determine the exact relationship between altered cholesterol absorption and age-related changes in the female jejunum.

The most important finding of this research related to the changes in cholesterol metabolism in the liver was likely the age-related increase in 27-hydroxycholesterol, seen only in female livers. 27-hydroxycholesterol is an oxysterol with multiple biological functions, mediated by tissue-specific modulation of estrogen and liver X receptors [54]. In addition, increased local concentrations of 27-hydroxycholesterol have been associated with various pathologies, including atherosclerosis, certain cancers, and neurodegenerative diseases [55]. Notably, this is the first identified endogenous selective estrogen receptor modulator (SERM) associated with the loss of protective effects of estrogen against vascular disease [56,57]. The putative role of 27-hydroxycholesterol in the development of liver and metabolic diseases is still unclear, although its concentrations in serum correlate directly with those of cholesterol and increase progressively with age in humans [54]. It is reasonable to speculate that under our experimental conditions, the SERM effect of 27-hydroxycholesterol may be potentiated by the increased expression of ER observed in the livers of older females. Long-term therapy with tamoxifen, a SERM used to treat breast cancer, has been associated with the development of fatty liver, steatohepatitis, and cirrhosis [57].

CYP27A1 is the only enzyme responsible for converting cholesterol to 27-hydroxycholesterol via the alternative pathway of cholesterol degradation to bile acids in mitochondria. Unlike CYP7A1, which is mainly found in the liver and catalyzes the initial and rate-limiting step in the primary pathway of cholesterol degradation to bile acids, CYP27A1 is an enzyme found in various tissues and can hydroxylate cholesterol and other sterols [35]. Our results regarding gene expression and CYP27A1 immunostaining in aged females provide limited evidence for enzyme induction. Alterations in cholesterol transport to mitochondria could potentially affect substrate availability, as this process is considered the rate-limiting step in the alternative pathway of cholesterol degradation to bile acids [58].

## 4. Material and Methods

### 4.1. Animals

Intact young adult (4-month-old) and aged (24-month-old) male and female Wistar rats were used for this research (*n* = 6/group). The animals were bred and housed in the Unit for Experimental Animals at the Institute for Biological Research “Siniša Stanković”—National Institute of the Republic of Serbia (IBISS), Belgrade, Serbia. All animals were kept under controlled light (12 h light–12 h dark) and temperature conditions (21 ± 2 °C). The animals were fed a normal grain-based pellet diet (IG-Z-00117, Gebi d.o.o., Čantavir, Serbia) containing: 86.5% dry matter, 20.3% protein, 3.2% fat, 49% carbohydrate (2.6% sugar), 12.9% fiber, 8% ash, and 1405 kJ (333 kcal)/100g energy. Food and water consumption were available ad libitum.

The experimental procedures were conducted according to the ethical guidelines for the care and use of laboratory animals. The animal procedures were in accordance with Directive 2010/63/EU on the protection of animals used for experimental and other scientific purposes and were approved by the Ethical Committee for the Use of Laboratory Animals of IBISS, University of Belgrade (No 2-12/12).

The animals were decapitated at the appropriate age without anesthesia. Before decapitation, the 24-month-old females were acyclic, in constant estrus or diestrus, as determined by vaginal smear analysis. Their ovaries had limited numbers of corpora lutea and antral follicles, suggesting occasional or sporadic ovulation.

Two animal experiments were performed independently (*n* = 6/group each time), and similar results were obtained.

### 4.2. Collection of Serum, Liver, and Small Intestine Tissues

Blood was collected from the trunk, centrifuged at 3000 rpm for 20 min at room temperature, and the separated serum was stored at −80 °C. The liver was perfused in cold physiological saline, then collected and measured. Relative liver weight was calculated for each animal as the percentage of absolute liver weight to total body mass. Small intestine samples (jejunum and ileum) were rapidly rinsed in cold physiological saline and then further processed either for tissue histology or stored after freezing in liquid nitrogen at −80 °C for other analyses.

### 4.3. Histological, Immunohistochemical, and Morphometric Analyses

Samples of the liver were collected and fixed in Bouin fixative for 48 h, while samples of the jejunum were fixed in neutral buffered formalin (10%, pH 7) for 24 h. After fixation, the tissues were dehydrated in an ascending series of ethanol (30–100%) and embedded in Histowax^®^ (Histolab Product AB, Gothenburg, Sweden). Four to five cross-sections of the liver and small intestine per animal (*n* = 6/group) were stained with hematoxylin and eosin (H&E) and used for further histopathological and morphometric analyses. All digital images were obtained using a LEITZ DM RB photomicroscope (Leica Mikroskopie & Systems GmbH, Wetzlar, Germany), with a Leica DFC 320 CCD Camera (Leica Microsystems Ltd., Heerbrugg, Switzerland) and LeicaDFC Twain Software v. 7.3, (Leica, Germany) for image acquisition and analysis, respectively.

Morphometric analysis of the liver and small intestine (jejunum) was performed on five randomly selected H&E-stained cross-sections per animal (*n* = 6/group) using a new CAST stereological software package (VIS–Visiopharm Integrator System, version 3.2.7.0; Visiopharm; Denmark).

A test grid (6 × 6), with uniformly spaced test points and lines, was used for histomorphometric assessment of the relative volume densities (V_V_) of the parenchyma (hepatocytes) and stroma (portal vessels, central veins, sinusoids, portal tracts, bile ducts, and others) at 20× magnification of the microscope. Briefly, after defining the tissue boundaries (low objective magnification 4×), meander sampling was adjusted to analyze 30% of the tissue using the software CAST. The V_V_ of the above tissue components was calculated as the ratio of the number of points hitting the corresponding tissue phase divided by the number of points hitting the entire reference space, the sum of all points: V_V_ (%) = Pp/Pt × 100 (Pp, counted points hitting the corresponding tissue component; Pt, a total of points of the test system hitting the reference space, the sum of all counts).

For liver immunohistochemistry, the procedure was applied exactly as previously reported [59]. Rabbit antiserum directed against CYP27A1 (1:100; Abcam, Cambridge, UK) was applied overnight at 4 °C. For immunodetection, a VECTASTAIN^®^ ABC (Rabbit IgG; Vector Laboratories, Newark, CA, USA) kit the biotin/avidin system was used according to the suggested procedure. All washes and dilutions were performed using 0.1 mol/L PBS pH 7.2. Hematoxylin was used as a counterstain, and the slides were then mounted in DPX medium (Sigma-Aldrich, Madrid, Spain).

For the quantification of the DAB IHC signal, we used Windows-based ImageJ Fiji software (ImageJ, Version 1.49j) with the open-source plugin IHC profiler [60], exactly as previously reported [59]. Optical density (OD) was calculated as OD = log (max intensity/mean intensity). For the analysis, six randomly captured images (2088 × 1550 pixels 40× objective magnification) per liver tissue per animal were analyzed.

For histomorphometric analysis of the H&E sections of the jejunum, the villus height, epithelium height, and crypt depth were measured using a linear distance measurement tool from the CAST software. For measurement of villus height, 12–15 villi were measured from their base at the level of the crypt’s entrance through to their distal tips. Only full and well-oriented villi were examined. For the determination of epithelium height, 30 epithelial cells of different villi were measured from the basement membrane to the top of their microvilli. For the crypt depth determination, 12–15 crypts were measured from the crypt’s base to the closest villus base. The ratio of villus height to crypt depth was calculated by dividing the villus height by the crypt depth. Further histomorphometric examination of jejunal mucosa included determination of the relative volume density of parenchyma (enterocytes plus goblet cells) and stroma, also using a test grid (6 × 6) with uniformly spaced test points and lines at an objective magnification of 20×.

### 4.4. Quantification of Sterols and Oxysterols in Serum, the Liver, Jejunum, and Ileum

The serum samples were carefully thawed, and 100 µL was used for sterol and oxysterol analysis [61]. Aliquots of liver and intestinal tissue samples were dried in a Speedvac concentrator (Savant DNA120, Thermo Scientific GmbH, Karlsruhe, Germany) at room temperature and the dry weight was taken as the basis for calculation of the sterol and oxysterol concentrations. Sterols and oxysterols were extracted using chloroform/methanol (2:1) and an aliquot of 100 µL of the sterol extract was used for further work-up as described in [62]. Cholesterol, the non-cholesterol sterols (cholesterol precursors: lanosterol, desmosterol, and lathosterol), as well as the plant sterols: campesterol, sitosterol, and stigmasterol and cholesterol degradation products: 7α-, 27- and 24-hydroxycholesterol, were quantified in order to describe cholesterol metabolism in serum, the liver, jejunum, and ileum exactly as previously reported [62].

The mono-trimethylsilylated sterol and di-trimethylsilylated oxysterol ethers were separated by gas chromatography from the same lipid liver extract or serum sample. Cholesterol was detected by less sensitive flame-ionization detection (FID) (5α-cholestane, internal standard, ISTD), and the non-cholesterol sterols (epicoprostanol, ISTD) and the oxysterols (2Hx-oxysterols, ISTD) were detected by highly specific and sensitive mass spectrometry in the selected ion monitoring mode (MS-SIM). The identity of all sterols was proven by comparison with the full-scan mass spectra of authentic compounds.

### 4.5. Determination of Oxidative Stress Biomarkers

The liver and jejunum samples were minced and homogenized in 10 volumes (liver) and 5 volumes (jejunum) of 25 mmol/L sucrose with 10 mmol/L Tris-HCl, pH 7.5, supplemented with 1×phosphatase inhibitor mix I and 1×protease inhibitor mix G at 1500 rpm using the IKA-Werk Ultra-Turrax homogenizer from Janke & Kunkel (Staufen, Germany) at 4 °C. The homogenates were sonicated on ice at 10 kHz for 30 s (Bandeline Sonopuls HD 2070) followed by centrifugation in a Beckman ultracentrifuge at 100,000× *g* for 90 min at 4 °C [63]. The supernatants obtained were used for the biochemical analyses. The activity of antioxidant defense enzymes was measured simultaneously in triplicate for each sample using a Shimadzu UV-1900i spectrophotometer and a temperature-controlled cuvette holder.

The activity of superoxide dismutase (SOD, EC 1.15.1.1) was determined by the epinephrine method [64]. One unit of SOD activity was defined as the amount of protein that causes 50% inhibition of the autoxidation of epinephrine to adrenochrome at 26 °C and was expressed as U/g wet mass. Catalase (CAT) activity (EC 1.11.1.6) was evaluated by the decomposition rate of hydrogen peroxide [65] and expressed as U/g wet mass where one unit is defined as µmol H_2_O_2_/min/g wet mass. The activity of glutathione peroxidase (GSH-Px, EC 1.11.1.9) was determined by following the oxidation of nicotinamide adenine dinucleotide phosphate (NADPH) with t-butyl hydroperoxide [66] and expressed as U/g wet mass, with one unit defined as nmol NADPH/min/g wet mass. The activity of glutathione reductase (GR, 1.8.1.7) was measured by the method based on the ability of GR to catalyze the reduction of oxidized glutathione (GSSG) to reduced glutathione (GSH) using NADPH as a substrate in phosphate buffer (pH 7.4) [67]. GR activity was expressed as nmol NADPH/min/mg protein. The activity of glutathione S-transferase (GST, EC 2.5.1.18) towards 1-chloro-2,4-dinitrobenzene (CDNB) was expressed as nmol GSH/min/mg protein. The method is based on the reaction of CDNB with the SH group of GSH catalyzed by GST present in the samples. Total glutathione (GSH) content was measured, as well as the concentration of the sulfhydryl (SH) groups being measured according to the method of [68]. The concentration of thiobarbituric acid reactive substances (TBARS) was estimated according to the method of [69]. The products of TBARS formed spontaneously, and the red color was formed by the reaction of thiobarbituric acid (TBA) with lipid peroxidation products (malondialdehyde). The measurement was performed after the samples were treated with cold thiobarbituric acid reagent (40% trichloroacetic acid and 0.6% thiobarbituric acid) and then heated at 100 °C and recorded at 532 nm. The LPO results were expressed as nmol TBARS/g tissue. The protein carbonyl concentration was determined according to the method of [70].

### 4.6. Gene Expression Analyses

Total RNA from the livers (*n* = 6/group) was isolated using TRIzol (Invitrogen, Carlsbad, CA) following the manufacturer’s instructions. Volume equivalent to 1 µg of RNA was used for reverse transcription to generate cDNA using a High-Capacity cDNA Reverse Transcription Kit (Applied Biosystems, Vilnius, Lithuania). Quantitative real-time PCR (qRT-PCR) was performed to evaluate the mRNA expression level, in a real-time PCR machine ABI Prism 7000 (Applied Biosystems, Waltham, MA, USA) with SYBRGreen PCR master mix (Applied Biosystems, USA), exactly as previously reported [20]. The sequences of the oligonucleotides designed using PrimerBLAST (NCBI) were: AR f: 5′-ACC CTC CCA TGG CAC ATT TT-3′, r: 5′-TTG GTT GGC ACA CAG CAC AG-3′; Cyp2c11 f: 5′-TGG AAG GAG ATC CGG CGT TT, r: 5′-GGG GCA CCT TTG CTC TTC CT; Cyp2c12 f: 5′-ACT TCA TAC CCA AGG GAA CAG CAG-3′, r: 5′-AGG CCC TCT CCC ACA CAT TTC-3′; Cyp27a1 f: 5′-ATG TGG CCA ATC TTC TCT ACC-3′, r: 5′-GGG AAG GAA AGT GAC ATA GAC-3′; Dio1 f: 5′-TTT AAG AAC AAC GTG GAC ATC-3′, r: 5′-GGT TTA CCC TTG TAG CAG ATC CT-3′; Erα f: 5′-GCGCAAGTGTTACGAAGTGG-3′; r: 5′-AGTGCCCATTTCATTTCGGC-3′; Hprt f: 5′-GCGCAAGTGTTACGAAGTGG-3′, r: 5′-AGTGCCCATTTCATTTCGGC-3′; Tbg f: 5′-AGGAAGGGCACATGGAATGG-3′, r: 5′-ACAGCCTTGTGAAAAGCATAGG-3′; Spot14 f: 5′-CTT ACC CAC CTG ACC CAG AA-3′; r: 5′-CAT CGT CTT CCC TCT CGT GT-32032. Each sample was tested in duplicate, and relative gene expression levels were analyzed by the 2^−ΔΔCt^ method. RNA data are presented as the average relative levels versus Hprt.

### 4.7. Serum Measurements of Biochemical Parameters

Serum concentrations of alanine (ALT) and aspartate (AST) aminotransferase were measured spectrophotometrically with appropriate diagnostic kits on a Roche Cobas 6000 (c501) automated analyzer (Roche Diagnostics, Mannheim, Germany). For the estimation of lipid profile, total cholesterol was determined by an enzymatic method), as well as HDL-C (a direct method of accelerator selective detergent) on Abbott Alinity ci-series (Abbott Diagnostics, Abbott Park, IL, USA), while non-HDL-C was calculated as total cholesterol minus HDL.

### 4.8. Statistics

The data were analyzed using the statistical program GraphPad Prism v.8 for Windows (San Diego, CA, USA). Two-way ANOVA analysis was used to analyze the effects of age and sex, as well as the interaction between the factors, on each tissue or serum sample. When a statistically significant effect was observed, Tukey’s HSD (honestly significant difference) post-hoc test was used to evaluate the differences between the groups. Differences were considered statistically significant at * *p* < 0.05, ** *p* < 0.01, *** *p* < 0.001, and **** *p* < 0.0001, with 95% confidence intervals. All results are expressed as the mean ± standard error of the mean (SEM).

## 5. Conclusions

Aging itself contributes to an increase in serum cholesterol concentrations in rats of both sexes. The age-related increase in serum cholesterol was more pronounced in females and was associated with poorer hepato-intestinal health and increased cholesterol in the jejunum and plant sterols in serum. Altogether, these data point to increased intestinal absorption as the main mechanism in female animals. A local age-related increase in 27-hydroxycholesterol and upregulated ERα gene expression were observed specifically in the livers of aged females. These changes may underlie the attenuated hepatoprotective effect of estrogen in the liver of old-aged females. Further studies are needed to explore the potential impact of these changes, which may be responsible for the attenuated hepatoprotective effect of estrogen in the livers of old-aged females. In the context of translational research, these findings could help develop personalized, sex-specific prevention and treatment approaches.

## Figures and Tables

**Table 1:** Parameters of the antioxidant defense system and oxidative-stress-induced damage in the rat liver.

LIVER				
	Male		Female	
	4 Months Old	24 Months Old	4 Months Old	24 Months Old
**SOD**	4229.21 ± 238.03	3422.69 ± 148.96 *	5240.94 ± 141.14 ^**#**^	4067.30 ± 496.44 *
**CAT**	45,205.96 ± 3565.04	56,429.24 ± 5534.05 *	68,532.40 ± 6549.49 ^**#**^	40,356.88 ± 1236.01 *^#^
**GSH-Px**	110,017.7 ± 7828.8	106,351.8 ± 2635.98	162,364.9 ± 17,537.1 ^**#**^	141,762.0 ± 2812.06 ^**#**^
**GR**	4085.2 ± 423.71	6753.10 ± 676.68 *	6967.84 ± 696.30 ^**#**^	7321.54 ± 552.21
**GST**	186,862.5 ± 18,052.3	177,522.3 ± 12,200.20	139,906.3 ± 6629.87 ^**#**^	159,316.7 ± 17,128.6
**GSH**	5770.6 ± 144.92	4679.30 ± 366.86 *	2870.46 ± 195.63 ^**#**^	3087.54 ± 119.97 ^**#**^
**SH**	3990.14 ± 189.64	3968.20 ± 241.74	3292.61 ± 201.85	4076.74 ± 153.81 *
**LPO**	11.53 ± 0.47	11.52 ± 1.01	13.99 ± 0.40 ^**#**^	13.71 ± 0.30 ^**#**^
**PCO**	4791.26 ± 428.16	3469.29 ± 202.72 *	3468.53 ± 74.29 ^**#**^	3672.75 ± 133.57

Activities of superoxide dismutase (SOD, U/g), catalase (CAT, U/g), glutathione peroxidase (GSH-Px, U/g), glutathione reductase (GR, U/g), and glutathione S-transferase (GST, U/g) and the concentrations of total glutathione (GSH, nmol/g), sulfhydryl groups (SH, μmol/g), lipid peroxides (LPO, nmol/mg), and protein carbonyls (PCO, μmol/g) in the livers of the 4- and 24-month-old male and female Wistar rats. The data are presented as the mean ± SEM, *n* = 6/group; * *p* < 0.05 for 4-month-old vs. 24-month-old rats; **^#^** *p* < 0.05 male vs. female rats of the same age, two-way ANOVA followed by Tukey’s test.

**Table 2:** Parameters of the antioxidant defense system and oxidative stress-induced damage in the rat jejunum.

JEJUNUM				
	Male		Female	
	4 Months Old	24 Months Old	4 Months Old	24 Months Old
**SOD**	970.91 ± 71.62	998.08 ± 70.66	1055.48 ± 88.14	1139.35 ± 79.6
**CAT**	266.87 ± 40.36	270.18 ± 55.99	821.97 ± 87.25 ^**#**^	525.69 ± 52.75 *^#^
**GSH-Px**	3077.86 ± 277.89	2305.47 ± 165.04 *	1895.50 ± 147.60 ^**#**^	1823.15 ± 372.38 ^**#**^
**GR**	5149.06 ± 445.14	3040.19 ± 409.55 *	5810.93 ± 377.83	4987.14 ± 332.52 *^#^
**GST**	17,394.64 ± 1624.05	11,041.67 ± 736.54 *	14,172.50 ± 1531.93 ^**#**^	12,312.50 ± 934.77 *
**GSH**	543.94 ± 70.50	600.45 ± 33.48	804.87 ± 12.90 ^**#**^	936.13 ± 76.11 ^**#**^
**SH**	1194.24 ± 90.95	1118.26 ± 119.23	966.66 ± 49.49 ^**#**^	818.52 ± 13.49 *^#^
**LPO**	6.84 ± 1.11	8.68 ± 1.22	7.52 ± 0.37	7.79 ± 0.07 ^**#**^
**PCO**	1462.00 ± 84.22	1172.97 ± 79.44 *	1313.07 ± 54.53	1278.91 ± 55.68

Activities of superoxide dismutase (SOD, U/g), catalase (CAT, U/g), glutathione peroxidase (GSH-Px, U/g), glutathione reductase (GR, U/g), and glutathione S-transferase /GST, U/g) and the concentrations of total glutathione (GSH, nmol/g), sulfhydryl groups (SH, μmol/g), lipid peroxides (LPO, nmol/mg), and protein carbonyls (PCO, μmol/g) in the jejunum of the young adult and old-aged (4 and 24 months old, respectively) male and female Wistar rats. The data are presented as the mean ± SEM, *n* = 6/group; * *p* < 0.05 for the 4-month-old vs. 24-month-old rats; **^#^** *p* < 0.05 male vs. female rats of the same age, two-way ANOVA followed by Tukey’s test.

## Data Availability

The datasets used and analyzed during the current study are available from the corresponding author on reasonable request.

## Supplementary Materials

The supporting information can be downloaded at: https://www.mdpi.com/article/10.3390/ijms241612624/s1.
