# Supplementary material for: Differences in Cholesterol Metabolism, Hepato-Intestinal Aging, and Hepatic Endocrine Milieu in Rats as Affected by the Sex and Age
Source: Int J Mol Sci. 2023 Aug 10;24(16):12624. doi: 10.3390/ijms241612624 (PMC10454938; doi:10.3390/ijms241612624)
Supplement: Supplementary file 1 [file ijms-24-12624-s001.zip › ijms-2548613-supplementary.pdf]

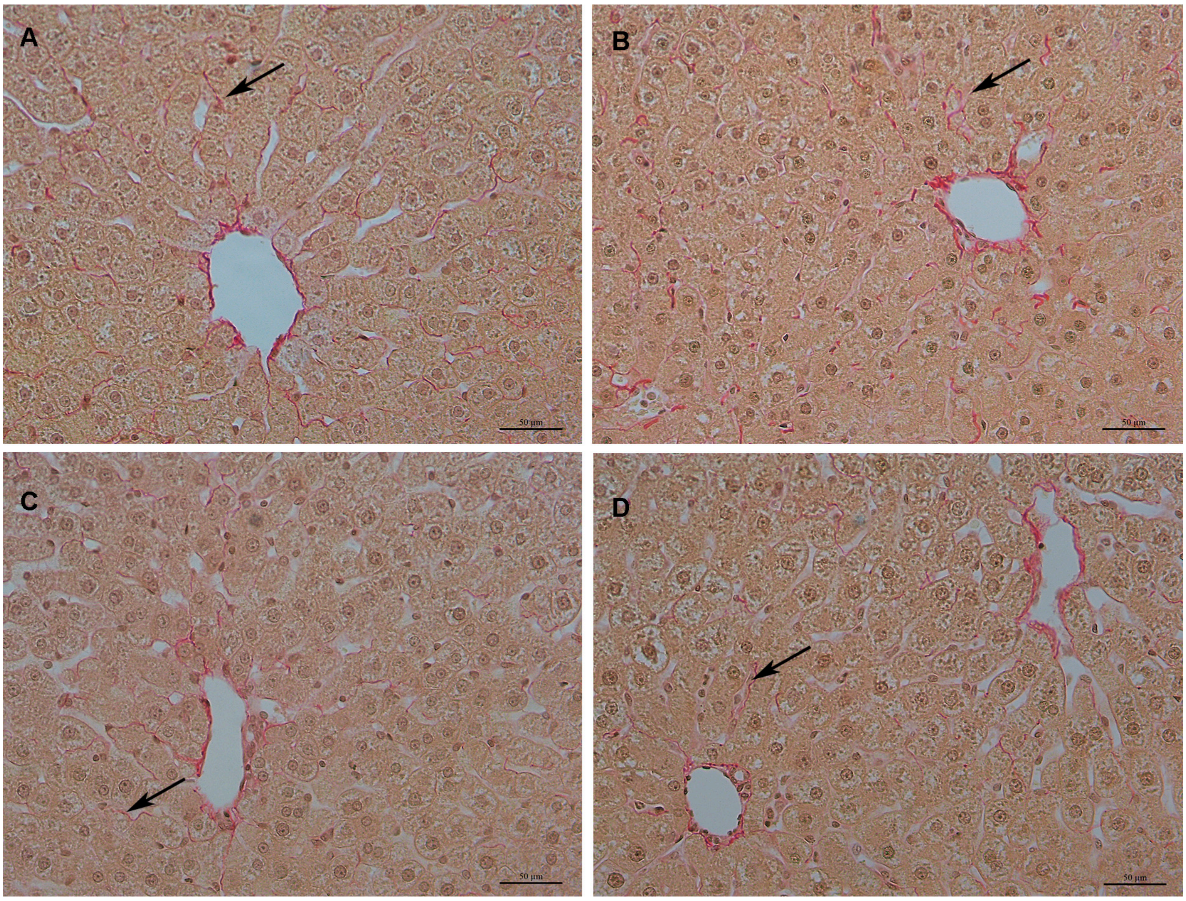

Figure S1. Representative micrographs of Sirius Red-stained liver sections from young (4-month-old) and old-aged (24-month-old) male (**A**,**B**) and female (**C**,**D**) rats, respectively; black arrows point on collagen fibers.
